# A Bio-Economic Evaluation of Var, LnVar, and r-Auto Resilience Indicators in Czech Holstein Cattle

**DOI:** 10.3390/ani15243593

**Published:** 2025-12-14

**Authors:** Zuzana Krupová, Eva Kašná, Ludmila Zavadilová, Emil Krupa

**Affiliations:** Institute of Animal Science, Přátelství 815, Uhříněves, 104 00 Prague, Czech Republic; kasna.eva@vuzv.cz (E.K.); zavadilova.ludmila@vuzv.cz (L.Z.); krupa.emil@vuzv.cz (E.K.)

**Keywords:** dairy cattle, health, welfare, production, reproduction, longevity, profitability

## Abstract

Farming animals that are resilient to various instabilities could improve both animal welfare and system sustainability. We evaluated three resilience indicators (Var, LnVar, and r-auto) in Holstein cattle on Czech farms using a bio-economic approach. We considered 3655 cows based on their genetic predisposition as 25% most resilient (Q3), median (Q2), and 25% least resilient (Q1), as well as their performance characteristics from routine production testing. Most of the performance characteristics significantly differed (*p* < 0.05) among the defined resilience quartiles. Q3 cows had slightly lower milk yield, higher milk component content, better udder health, and shorter dry periods compared to Q2 cows. The longevity of Q3 cows differed according to the indicator used (this was higher in Var and LnVar, but lower in r-auto). The highest profitability was found for the Q2 group in Var and r-auto and for Q1 in LnVar. Across all three resilience indicators, Q3 cows were the least profitable. Milk yield, lactation persistence, longevity, and milk fat and protein content contributed most to farm profit change across the resilience groups. The generality and simulation accuracy confirmed that the bio-economic model is suitable for the comprehensive setting and economic evaluation of resilience indicators and cattle performance.

## Simple Summary

Resilience describes an animal’s capacity to cope with environmental perturbations and rapidly return to its previous status. This study provides a comprehensive evaluation of three resilience indicators in Czech Holstein cattle: log-transformed variance of daily milk yield (Var), log-transformed variance (LnVar), and lag-1 autocorrelation (r-auto) of daily deviations from predicted lactation curves. The results display the broad impact of animal resilience indicators on herd structure, production, reproduction, costs, revenues, and overall farm economics. The findings of our study support the general benefits of resilience, revealing the biological specifics of indicators involving milk yield trade-offs. Using a bio-economic approach, performance trait variability among resilience groups was reflected in economic terms. Our data investigation and the model’s general construction revealed further relationships and system connections to consider in cases where relevant data are available. Proper information and resilience analysis could advance the sustainability of local Holstein farms currently operating close to breaking even. Animals with an average resilient indicator maintain their performance and associated profitability, which could represent an optimal balance. These findings can be implemented into daily management strategies on dairy farms for animal selection and environmental stability.

## 1. Introduction

Livestock production faces many contemporary challenges relating to both animal welfare and long-term system sustainability. In this context, farming the most resilient animals could play an important role. Resilience is described as “the capacity of animals to cope with short-term perturbations in their environment and return rapidly to their pre-challenge status” [1], and this quality can be improved through genetic and management strategies [2]. Resilience benefits farms through improved animal health, reduced treatment times, and more stable production [1,2].

Sources of instability could include various pathogens, diseases [3], heat stress, physical and social stressors, and any general perturbations in living conditions [4]. Because information about environmental instabilities is usually insufficient, some time series fluctuation-based indicator variables have been suggested to define resilient animals [1,5]. Animals that were less affected by disturbances showed fewer fluctuations and were therefore considered more resilient compared to those with more fluctuating performances [2,6]. The variance in daily milk yield (DMY) during lactation was shown to be heritable and genetically associated with fertility, health, and longevity in dairy cows [5]. The authors of [5] did not account for lactation curve shape, which influences milk yield variance. Therefore, several metrics have been investigated to quantify resilience based on daily deviations in production traits, activity, or different predicted performance biomarkers [2,4,6,7,8]. In dairy cattle, LnVar and r-auto have promising heritability levels and sufficient accuracy of genetic estimates and show favorable relationships with functional traits and body condition scores [4,9]. They also contain information on different aspects of resilience: lower LnVar corresponds to animals without disturbances or unaffected by them, while r-auto around zero, i.e., with unrelated subsequent deviations, indicates animals that were not influenced or that recovered from disturbances quickly [2,6,8]. Nevertheless, their relationship with milk yield is usually unfavorable, as high-production cows are observed to have greater milk yield variability [7,9]. Resilience should therefore be evaluated while taking into account its biological complexity [1], the indicators used as descriptors [2], and its relationship with other animal performance traits (e.g., [4]).

In economic terms, the impact of resilience has been evaluated using simple profit functions while taking into account reduced labor and disease treatment costs with more resilient dairy cows [2]. In an economic evaluation of specific disease resilience, including its impact on potential performance, a relatively complex approach based on reaction norm models was implemented [3]. The calculations in both studies were conducted for breeding (selection) purposes, purely considering the direct economic effects of resilience (and its components). Nevertheless, from a farmer’s perspective, and in accordance with the above-mentioned studies, resilience may have broader economic effects. Bio-economic models may generally provide appropriate and comprehensive economic evaluations regarding the biological aspects of production systems [10]. To the best of our current knowledge, no comprehensive economic evaluation embracing a spectrum of productive and reproductive parameters according to the respective resilience indicators has yet been carried out in the literature. An analytical tool of this nature could help farmers to understand the consequences of resilience in day-to-day management decisions. Moreover, a deeper recognition of resilience indicators’ complexity and associated performance could be beneficial.

The main aim of our study was to therefore provide a comprehensive economic evaluation of three resilience indicators, which were selected based on the above literature and our previous analyses [9]. For this purpose, we broadly considered the productive and reproductive parameters for Czech Holstein cattle using a bio-economic approach. In this sense, we hypothesized that the national lifetime performance database represents a useful source of information regarding the specific characteristics for evaluating animal resilience. Secondly, the general construction of a bio-economic model could enable us to evaluate such parameters comprehensively in economic terms.

## 2. Materials and Methods

The comprehensive economic effect of resilience indicators was determined through phenotypic and genetic parameters recorded for Czech Holstein cattle by means of bio-economic evaluation. Detailed definitions of genetic parameters, as well as the genetic trait evaluation methodology, have been described in a previous study [9]. Likewise, the basic biological (e.g., production, reproduction, and health) and economic (costs, revenues, profit, and profitability) characteristics of Czech Holstein cows, together with the principles of the program EWDC bio-economic model [11], have also already been published in detail [12,13]. Therefore, only some specifics regarding the initial dataset, additional phenotypic data (regarding animal performance and herd structure), and the specific economic evaluation considered in the current study will be presented in the following text.

### 2.1. Initial Dataset and Resilience Indicator Quartiles

The initial dataset contained 1,776,254 daily milk yields recorded on 10 farms between May 2022 and July 2024. The farms were equipped with DeLaval milking robots (AMS, 7 farms), conventional Afimilk milking parlors (2 farms), or a GEA automatic rotary milking parlor (1 farm). Data manipulation, editing, and analyses were performed using SAS/STAT software, v9.4 (SAS Institute, Inc., Cary, NC, USA). The AMS records were adjusted to daily 24 h yields according to [14] to avoid any additional variation from irregular milking station visits. DMY records were discarded when they were <2 kg or >99 kg. Lactations from non-Holstein cows were excluded (a breed proportion of at least 75%), as were lactations with days in milk (DIM) < 0 or DIM > 350 or with less than 100 DIM recorded. The final dataset contained 1,160,219 DMY records on 3971 lactations (Table 1). We used Ali and Schaeffer’s model [15] (a third-order polynomial) to fit the lactation curve. The regression coefficients for each cow were estimated using PROC REG (SAS/STAT) and used to calculate the individual lactation curves. Resilience indicators were calculated as the log-transformed variance of DMY (Var), log-transformed variance of daily deviations (LnVar), and lag-1 autocorrelation of daily deviations (r-auto) within each cow and lactation.

The systematic effects (herd, year and season of calving, parity) were analyzed using the generalized linear model method, PROC GLM (SAS/STAT). Variance components and genomic breeding values for resilience indicators were predicted with a set of single-trait animal repeatability models. Genomic evaluation was based on a single-step genomic prediction method (ssGBLUP), as implemented in the BLUP90 family of programs [16]. The animal model equation was as follows:

*y_ijklm_* = *µ* + *HYS_i_* + *Par_j_* + *a_k_* + *pe_l_* + *e_ijklm_*, (1)

where *y_ijklm_* is the evaluated resilience indicator, i.e., Var, LnVar, or r-auto; *μ* is the population mean; *HYS_i_* is the fixed effect of herd–year–season *i* (40 classes); *Par_j_* is the fixed effect of parity *j* (three classes, 1st, 2nd and 3rd, and later); and *a_k_* is the random effect of individual *k* joined with three-generation pedigrees (12,527 classes) supplemented with SNP 50K Illumina bovine genotypes for 2078 animals. After quality control, we used 37,367 SNPs and 1994 animals to construct a genomic relationship matrix using the BLUP90IOD program. Furthermore, in the equation, *pe_l_* is the random effect of the permanent environment of a cow *l* (3673 classes), while *e_ijklm_* is the random residual.

Genomic breeding values were expressed as relative breeding values (RBVs) with mean = 100 and standard deviation (SD) = 12 for base bulls born in 2010:

*RBV* = (*GEBV* − *mean of the genetic base/SD*) × 12 (2)

The RBVs were then reversed; this is because higher values are desirable, corresponding with less variable performance, faster recovery, and therefore genetic predisposition for better resilience.

Data on actual production, reproduction, and functional traits from the national lifetime performance database were available for 3655 cows with predicted RBVs. Based on their RBV quartiles, and separately for each indicator, these cows were classified as 25% most resilient (upper quartile, Q3), median (Q2), and 25% least resilient (lower quartile, Q1). Descriptive statistics for the evaluated data are presented in Table 1.

### 2.2. Animal Performance and Herd Structure

**Figure 1:**
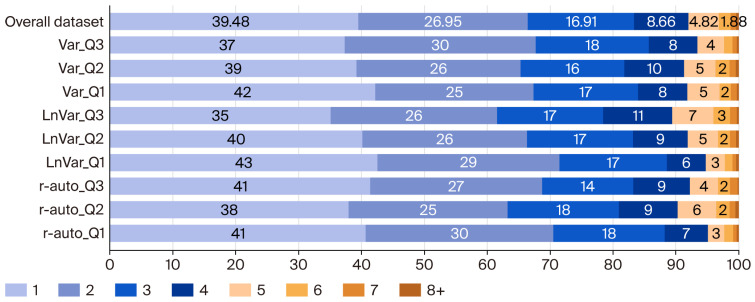
Cow herd structure ^1^ for the overall dataset and resilience indicator quartiles ^2^. ^1^ Expressed as cow distribution (in %) by lactation number (from the first to eighth lactation). Lactation frequency ≤ 2% was not labeled here. ^2^ Quartiles: Q3—25% most resilient cows; Q2—median; and Q1—25% least resilient cows, according to their relative breeding values estimated for the following resilience indicators: Var—natural log-transformed variance of daily milk yield; LnVar and r-auto—natural log-transformed variance and lag-1 autocorrelation of daily deviations of observed milk yields from the predicted individual lactation curve, respectively. Based on the authors’ calculations from the database provided by CMBC, Inc.

The Czech and Moravian Breeders’ Corporation (CMBC, Inc.) provided animal data from the national lifetime performance database. Performance parameters included milk yield over a 305-day milking period (MY), fat and protein production in the same period (FY and PY), milk somatic cell count (SCC), lactation persistence (PER), days in lactation (LD), insemination interval (II), service period (SP), calving interval (CI), age at first calving (AFC), age of cows at culling (CUL), and longevity (LONG). An overview of the evaluated phenotypic parameters and their basic characteristics—according to the Var, LnVar, and r-auto quartiles—as well as the overall dataset is presented in Table 2. Cow frequency distributions by lactation number (herd structure), as calculated for the overall dataset and for the individual resilience indicator quartiles studied, are shown in Figure 1.

**Figure 2:**
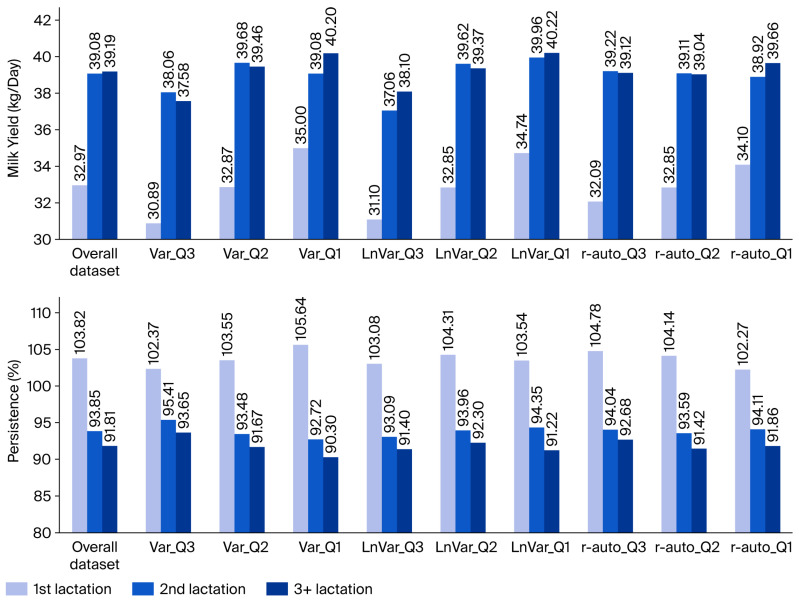
Average daily milk yield ^1^ and lactation persistence ^2^ among cows in respective lactations for the overall dataset and resiliency indicator quartiles ^3^. ^1^ Calculated as daily mean (kg) from milk yield per 305 days in milk. ^2^ Persistence defined as rate (%) of milk yield in the second versus first 100 days of lactation. ^3^ Quartiles: Q3—25% most resilient cows; Q2—median; and Q1—25% least resilient cows, according to their relative breeding values estimated for the following resilience indicators: Var—natural log-transformed variance of daily milk yield; LnVar and r-auto—natural log-transformed variance and lag-1 autocorrelation of daily deviations of observed milk yields from the predicted individual lactation curve, respectively. Mean values over lactations relevant for the cow in respective resilience indicator groups are presented in Table 2. Based on the authors’ calculations from the database provided by CMBC, Inc.

Cows’ lactation persistence and daily milk yield in subsequent lactations (for the first, second, and third and higher) were taken into account to consider the variability of traits across the dataset and within the resilience indicator quartiles (Figure 2). Persistence was defined as the rate (%) of milk yielded in the second versus first one hundred days of lactation. Milk yield (kg/day) corresponded to milk yield per 305 DIM, calculated from averages on individual test days and considering the numbers of days between these days.

Phenotypic data were checked for normality (PROC UNIVARIATE, SAS/STAT) and homoscedasticity (PROC GLM, PROC MIXED, SAS/STAT), while differences in phenotypes according to resilience classes were evaluated separately for each indicator. The model equation was as follows:

*y_ijk_* = *µ* + *HYS_i_* + *Par_j_* + *Q_k_* + *e_ijk_*, (3)

where *y_ijk_* is the phenotype, *μ* is the population mean, *HYS_i_* is the fixed effect combining 10 herds with 3 years and 3 calving seasons (40 classes), *Par_j_* is the fixed effect of parity (three classes, 1st, 2nd and 3rd, and later), *Q_k_* is the resilience class (three classes: Q1, Q2, Q3), and *e_ijk_* is the residual.

We used pairwise comparison of LS-means in PROC MIXED (SAS/STAT) for traits with normal distribution (milk, fat and protein yield, fat and protein content, SCS, persistency) and in PROC GLIMMIX (SAS/STAT) to evaluate right-skewed interval traits.

### 2.3. Bio-Economic Analyses

Distinguished by the three indicators (Var, LnVar, and r-auto), analyses of the impact of resilience groups on the economics of dairy farms were carried out using the EWDC program bio-economic model [11]. In the current study, we updated the production and economic parameters for Czech Holstein cattle, as applied in the model [12,13], to represent the overall dataset parameters and the resilience quartiles studied, as defined and described above. The methodological principles implied in the model—and associated with the main aim of this study—were applied for three tasks: examining milk production as the basis for deriving resilience indicators, comparing variability in cow herd structures among resilience groups, and performing an economic evaluation of resilience indicator groups.

The resilience indicators used in this study were built on the variance in daily milk yield or its deviation from the optimal lactation curve (i.e., residuals). In the bio-economic model, to simulate and describe the lactation curve and estimate lactation persistence, taking into account days in pregnancy, the Wood function [17], as modified by [18] (latterly evaluated by [19]), was used as follows:

(4) $$MP\left(t\right)=at^{b}\exp\left(-ct\right)\exp\left(-dp\right),$$

where *MP*(*t*) is the milk yield at day *t* of lactation, *a*, *b*, *c*, and *d* are parameters, and *p* is the number of days in pregnancy.

The parameter *a* was calculated from the average yearly milk production per cow (YMP, in kg [18]) as follows:

*a* = (0.01YMP − 20 × 0.454)/2.96 (5)

for the first lactation, and

*a* = (0.01YMP + 14 × 0.454)/2.96 (6)

for the second and subsequent lactations.

The constant 0.454, which is not contained in the original equations of [18], represents the conversion between pounds and kilograms.

The parameters *b*, *c*, and *d* were set to represent the average daily milk yield and lactation persistence already calculated from the performance testing database (Figure 2). In accordance with this database, lactation persistence was calculated as the rate (%) of milk yield in the second versus first 100 days of lactation, with milk yield being characterized as the average daily yield over a 305-day milking period. As an input for the bio-economic model, lactation persistence was expressed according to parameter estimates determined by the lactation curve mathematical model mentioned above. The relevant input values for the lactation curve parameters are presented in Table 3.

Somatic cell counts (SCCs) taken from the performance testing database represent the average number of somatic cells per milliliter of milk. For the economic evaluation, this score was expressed as follows [20]:

(7) $$SCS=log_{2}\left(\frac{SCCs}{100000}\right)+3$$

Similarly, milk fat and protein production (in kg/305 days, see Table 2) was expressed as a percentage for compatibility with the program’s input definition. Additionally, days in dry period and pregnancy length (days) were calculated from the performance testing data as program inputs relevant to herd turnover. Additional phenotypic parameters and their basic characteristics for the overall dataset and resilience quartiles are shown in Table 4.

For the resilience groups and overall dataset, cow herd structure was derived as the stationary state of a Markov chain using an iteration procedure and an iteration convergence criterion close to zero (as detailed in [11,21]). In terms of herd structure, the cow category was defined by the number of reproduction cycles (eight at maximum in the current study (see Figure 1)) and stages in a given cycle (i.e., cows that died, that were culled due to health, low milk production and no pregnancy, and entering the next reproduction cycle pregnant). In total, 39 cow categories were defined (7 × 5 + 4, with four stages in the last cycle).

The economic efficiency of the production systems, as represented by the resilience groups and overall dataset, was expressed firstly as the present total profit value (total revenues − total costs, in EUR/year) per cow entering a reproductive cycle, and secondly as profitability ratio (cost effectiveness, as profit × 100/total costs, in %). It was assumed that milk fat and protein content, along with SCS, influenced milk price. We assumed an average price of EUR 0.426 per kg of milk and the most common payment system in local dairies (described in [12]). Total revenues, costs, and economic efficiency were functions of milk amount and quality, herd structure, production, reproduction, and the nutritional parameters of all cattle categories [11,21]. Total costs included those for feeding, housing, veterinary treatment, dystocia, fixed (e.g., labor, depreciation, insurance, and housing), and other (e.g., removing and rendering dead animals and breeding costs). An exchange rate of CZK 25.119/1 EUR was also applied.

To evaluate the economics of the overall dataset and resilience indicator groups, performance parameter settings were progressively employed in the bio-economic model as follows:

Herd turnover—LD, II, SP, CI, days in dry period, and pregnancy length (mean, Table 2 and Table 4);
Age of heifers entering herds—AFC (mean, Table 2);
Lifetime—cow herd structure (Figure 1), CUL, and LONG (Table 2);
Udder health—SCS (mean and standard deviation, Table 4);
Milk quality—fat and protein content (mean and standard deviation, Table 4);
Milk production and persistence—parameters in Table 3 to represent phenotypes (Table 2, Figure 2).

The partial proportion of the given phenotype block and, finally, the overall effect of the evaluated resilience quartile were evaluated economically. Some of the performance parameters were directly set as input values (II, days in dry period, pregnancy length, SCS, fat and protein content, parameters of the lactation curves), and the remainder (SP, LD, CI, AFC, cow herd structure, milk production, and persistence) were obtained as outputs from the program setting (result file and CHECKD file). In total, about 43 values were updated in the program files, and about 40 runs (most of them to achieve the intended cow herd structure presented in Figure 1) were made to reach the proposed resilience group parameters. To verify the suitability of the bio-economic model for economic evaluation, the overall phenotypic data based on the performance testing database (typical for all 3655 cows), as well as on inputs and outputs from the EWDC program, were subjected to detailed analysis (as mentioned in Section 3.1). Nevertheless, the continuous critical analyses of program inputs and outputs were carried out when each performance parameter block for a given data group was settled. The consistency of the production database parameters, as well as of the input and output parameters of the bio-economic model in one block, was a condition for setting parameters in the following block. Similarly, the overall dataset parameters were studied against officially published data [22] to judge their representativeness of the local Holstein population.

## 3. Results

### 3.1. Overall Phenotype Data and Model Outputs

Table S1 summarizes the performance parameters of the overall dataset implemented in the bio-economic model’s input and output files (i.e., input–output mapping). Regarding herd turnover, for the overall dataset, program inputs linked to pregnancy length, insemination interval, and days in dry period resulting in calving interval, service period, and days in lactation values are as presented above for the performance testing data (Table 2 and Table 4).

The age of heifers entering herds was calculated in the model as a function of their growth intensity, minimum live weight at first mating (400 kg), and conception rate (59.3%, 57.6%, and 55.0%, respectively, after the first, second, and third inseminations). The latter two parameters were kept constant for all program runs and settings. Growth intensity was proportionally modified in the input file (0.799 kg per day from birth to the end of the rearing period and 0.779 kg per day from the end of this period to first mating) to determine heifer age at first calving (AFC; 751.4 days), as already determined for the overall performance testing data. An absence of further resilience group-specific details (i.e., heifer conception rate and live weight) was the main reason for taking this approach. Nevertheless, in the case of data availability, such information can be applied and economically assessed within the bio-economic model.

Cow herd structure and associated lifetime were expressed as a function of various-cause losses within the given reproduction cycle (see Table S1). Losses related to culling for general health were reported most frequently (48%), followed by culling for low milk production and reproductive disorders. The cow herd structure from the program matched well with that found for the overall dataset (Figure 1).

**Figure 3:**
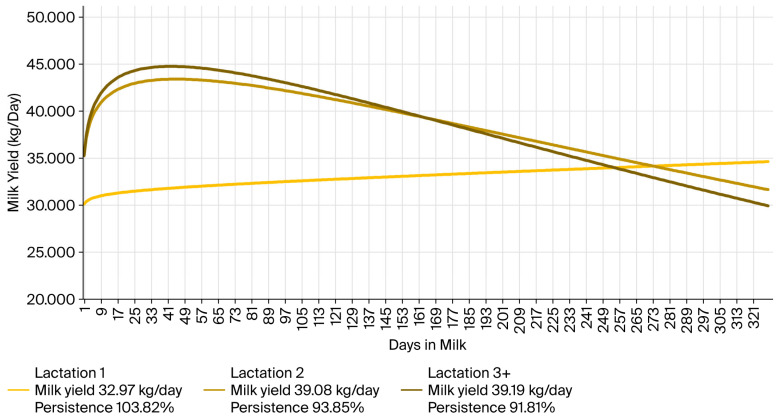
Lactation curves describing milk yield (kg/day) and lactation persistence (%) of cows in the overall dataset. Based on the authors’ calculations using the EWDC program [11] and the data provided by CMBC, Inc.

The following three parameter blocks were associated with milk production: SCS indirectly represented udder health, fat and protein content characterized milk quality, and lactation curve parameters defined milk yield and lactation persistence. Phenotype values and the parameters derived from the overall dataset (presented in Table 2, Table 3 and Table 4) were directly inserted into the input files (or calculated by program in the case of parameter *a*). The resulting lactation curves for cows in the first, second, third, and later lactations are presented in Figure 3. The same approach was applied for all resilience indicator quartiles. Daily milk yield and lactation persistence matched the performance data parameters (see Figure 2). Milk yield, as well as milk fat and milk protein production, per 305 days of lactation, as calculated in the program (see Table S1), corresponded to performance-testing data (with negligible differences of +0.37% to +1.04% (Table 2)).

### 3.2. Phenotypic Performance in Resilience Indicator Groups

Statistical differences between selected phenotypic parameter values for the studied resilience indicator quartiles are shown in Table 5. Most of the performance traits associated with milk production (fat and protein content, milk yield, SCS), herd turnover (insemination interval, length of lactation period), and longevity (age at culling and number of lactations) differed significantly (*p* < 0.05) among the resilience indicator quartile groups. Some significant differences (*p* < 0.05) were found for lactation persistence (Q2 vs. Q3 and Q1 vs. Q3, for LnVar; and Q1 vs. Q2 and Q2 vs. Q3, for r-auto) and age at first calving (Q2 vs. Q3 for r-auto). The results indicated that the performance database represented a useful source of information regarding characteristics for animal resilience evaluation. Defined quartiles were specific in most of the performance characteristics, with their evaluation thus being both reasonable and potentially valuable.

In terms of the performance characteristics calculated for the overall dataset and those for animals included in the median groups (Q2) (presented in Table 2 and Table 4), negligible differences were found. For example, milk yield per 305 days in the Q2 groups varied by −1 kg (in r-auto) to +69 kg (in Var indicator), respectively, representing 0% and 1% differences from the overall dataset values. Similar results were found for all performance traits, with an average difference of 1%. As observed in the Q2 group for the r-auto resilience indicator, SCC was the only trait with a slightly greater difference (5%) from the overall dataset value. However, this difference was more than halved (to 2%) when the logarithmic SCS value was used. Generally, the median performance trait values presented in Q2 groups and the average values calculated for the overall dataset were found to be very similar. In the following text, therefore, the quartiles Q3 and Q1 will be compared to the respective resilience indicator Q2 group, as well as to the overall dataset, to explore whether the two could substitute for one another.

Generally, more resilient cows (group Q3 in all indicators) had slightly lower milk yield (−3% on average), better milk quality and udder health (components content +2%, SCC −10%, and SCS −4%), and a shorter dry period (−8%) compared to Q2 cows, and vice versa in the case of less resilient cows (Q1 group). In terms of cow longevity, some Q3 group specifics were found. Cows more resilient according to Var and LnVar reached a higher age at culling and a higher number of lactations (+6% on average), whereas the longevity of Q3 groups was reduced when considering the r-auto resilience criterion (−8% on average). The most favorable values were seen in cows with median resilience (Q2 group with +109 days at culling and 0.22 lactations in relation to Q1 and Q3 cows on average). Very similar results were found when comparing the performance of more resilient cows (Q3) and the overall dataset mean (e.g., milk yield −3%, milk components content +2%, SCC −10%, and dry period −7%). Among the resilience indicators, the cow lifetime specifics corresponded to those of the Q2 groups mentioned above, demonstrating only slightly differing intensities (+5% vs. −6% on average for Q3 cows in LnVar and Var vs. r-auto criterion).

### 3.3. Resilience Economics on Dairy Farms

The economic parameters of the studied resilience groups, along with comparisons with the Q2 group (median) and overall dataset (mean), are summarized in Table 6. For the overall dataset, the annual farm profit was EUR 136 per cow, with profitability at 3.0%. The profitability of median resilience groups (Q2) was very similar (2.9% in LnVar) or slightly higher (3.4% in Var and r-auto (Table 6)).

Generally, the Q3 and Q1 resilience group profits deviated from the respective Q2 group more intensively than they did from the overall dataset (e.g., EUR 25 vs. 7 in Q1 group Var). This statement is supported by the significant differences seen in many performance traits among the respective resilience groups (as mentioned above and as can be seen in Table 5), which was probably transformed into profitability change (e.g. −0.5 vs. −0.2 p.p. for the Q1 group compared to the Q2 group in Var indicator vs. overall dataset, respectively (Table 6)). These results indicate that the identified quartiles described the performance variability in resilience indicators more specifically; however, they also imply that, in some instances, the overall dataset mean can be used as a reference.

The total annual revenues and costs per cow varied by, at most, ±1% in all of the data groups studied (Table 6). The resulting profit varied from EUR 115 (Q3 in Var) to 153 (Q2 in both Var and r-auto), meaning by −25% to +13%, respectively. This change was probably based on the absolute profit value, which was quite low (close to breaking even, as mentioned earlier). Therefore, any change in profit was likely to be more visible in relative terms. This assumption was supported by the farm profitability varying from 2.5% to 3.4% among the datasets studied (i.e., from −0.9 to 0.4 p.p.), which was consistent with changes in revenues and costs and indicated the model’s reasonable sensitivity.

Regarding the quartiles inside the respective resilience indicators, the highest profitability was found for the Q2 group in Var and r-auto (3.4% in both cases) and for the Q1 group in LnVar (3.3%). In contrast, the least profitable cows were in the Q3 groups for all three resilience indicators (2.5% to 2.8%). This reveals that, in more resilient cows (Q3), higher milk quality, better udder health, and shorter dry periods did not economically compensate for the lower milk yield (and in the case of the r-auto indicator, shorter lifetime (Table 2)). Moreover, cows with lower resilience (Q1) according to the LnVar indicator were able to fully reimburse losses from higher SCC in milk (+19%), longer dry periods (+4%), and shorter productive lifetimes (−14%) through their higher milk yields (+3%). The final farm profit exceeds the median (Q2) quartile by EUR 14 per cow and year.

**Figure 4:**
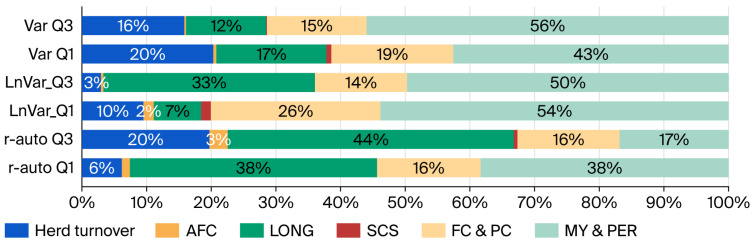
Proportions of phenotype parameter blocks ^1^ influencing overall profit change in the studied resilience groups ^2^. ^1^ Blocks: herd turnover (LD, II, SP, CI, days in dry period, and pregnancy length); AFC (age of heifers at first calving or entering herds), LONG (lifetime of cows—CUL, LONG; cow herd structure), SCS (somatic cell score; indirect parameter of udder health), FC and PC (fat and protein content; milk quality parameters), and MY and PER (milk yield and lactation persistence). For more detailed descriptions of parameters in blocks, see Table 2, Table 3 and Table 4, Figure 1 and Figure 2, and the Material and Methods section. ^2^ Groups (quartiles): Q3—25% most resilient cows; Q1—25% least resilient cows, according to their relative breeding values estimated for the following resilience indicators: Var—natural log-transformed variance of daily milk yield; LnVar and r-auto—natural log-transformed variance and lag-1 autocorrelation of daily deviations of observed milk yields from the predicted individual lactation curve, respectively. Overall profit difference in the studied resilience group from respective Q2 (median) values is presented in Table 6. Based on the authors’ calculations using the EWDC program [11] and the data provided by CMBC, Inc.

A partial proportion of the six phenotype blocks of the overall economic change in the analyzed resilience quartiles is presented in Figure 4. Generally speaking, milk yield and lactation persistence (43%), longevity (25%), and milk fat and protein content (18%) reached the highest contribution per change in farm profit over all Q3 and Q1 resilience groups.

## 4. Discussion

### 4.1. Phenotypic and Economic Parameters

A detailed investigation of the input and output data (as presented in Section 3.1) indicated the suitability of the EWDC program bio-economic model for setting performance parameters for economically analyzing resilience indicators. The Wood function was [17] implemented to simulate the lactation curve, and persistence is commonly used in the literature to describe lactation characteristics (particularly latterly, as seen, e.g., in [23,24,25]).

In terms of the data representativeness of the Czech Holstein population, the calving interval determined for our dataset (379.4 days) indicated a slightly better turnover compared with all Holstein cows included in performance testing (392 days [22]). The post-first mating heifer conception rate found in our data (59.3%) was close to the value (57.9%) published for all dairy breeds included [22]. When calculating the herd structure, health reasons were found to constitute the most frequent (60%) cause of dairy cow removal officially recorded [22]. In terms of daily milk yield and lactation persistence across all studied data groups, cows in first lactation typically had lower peak yield, lower total 305-day milk yield, and higher persistence compared to those in later parities (Figure 2). Similar results showing lower average milk yield and slower average decline in milk production in primiparous cows have been presented in the literature ([26]). Mean DMY per lactating cow across all parities (36.7 kg) was higher than the average milk yield (25.62 kg) published by CMBC [22]. The inclusion of non-lactating days (as MY was expressed per day of calving interval) and various breeds (e.g., Czech Fleckvieh and Montbeliard) in the official records was probably the main reason for this difference. In our evaluation, milk yield and fat and protein production per 305 days of lactation were close to the latest official breed records (10,743 kg of milk, 414 kg of milk fat [3.85%], and 362 kg of milk protein [3.37%] [22]). The overall dataset was found to be representative of officially recorded and reported characteristics; as such, we can presume that economic analyses of local Holstein populations are generally valid.

The profitability of the studied resilience groups (Table 6) corresponded to that most recently published for local Holstein farms (ranked from −13% to +12% [27]); it was also lower than the gross margin (32%) calculated for Swedish dairy farms [28]. Accounting for the fixed cost would additionally reduce the profitability found in this study. The slightly higher profitability of milk sold (11%) in Czech dairy farms published by the authors of [29] would probably be reduced when accounting for economics across the farm, such as when including all cattle categories (young heifers and fattened bulls), as we did in our study. Generally speaking, the profitability of local Holstein farms (found both in our study and in the above-mentioned literature) indicated that they operated slightly above breaking even and that any improvements in management strategies could have perceptible consequences for sustainability.

The average 1% performance variability across all traits and studied quartiles, as mentioned above (Table 2 and Table 4), was reflected in economic terms, becoming visible when comprehensive evaluation (e.g., using the bio-economic model) was applied. More intensive input parameter changes (20%) are usually applied in sensitivity analyses when using the simple profit function and holding other parameters constant (e.g., [27]). While these functions provide a basis for interpreting results on the relationship between animal performance and farm profit level, simulation methods (including the bio-economic approach) enable a more precise and flexible evaluation of different production systems and conditions through varying the model’s various input parameters [10]. Such a comprehensive approach was applied in our study.

Milk yield and lactation persistence, longevity, and milk fat and protein content represented the highest proportion in terms of profit change among the analyzed resilience quartiles (Figure 4). Accordingly, milk yield has been identified as one of the most important parameters influencing Czech dairy farm profits [27], followed by milk price and price of feed. Similarly, our study supported milk fat and protein content as the main determinants influencing milk price (Figure 4). Adamie et al. [28] and Bengtsson et al. [30] reported similar findings for the importance of longevity in farm economic performance.

### 4.2. Bio-Economic Evaluation of Resilience Indicators

The bio-economic evaluation of the three resilience indicators combines estimates of cows’ genetic predisposition to resilience with performance characteristics available (and derived) from routine production testing. Such a combination, as has been applied in other studies (e.g., by [28] in evaluating cow longevity), has been shown to be beneficial and yield valuable results. Although a relatively wide range of relationships and system connections was taken into account here, the rather complex impact of resilience on farm economics was revealed. We can therefore outline some areas for more detailed economic evaluation (in the event of sufficient data availability). A few of these relationships and connections have already been mentioned (e.g., heifer age at first calving). The biology of a respective resilience indicator was transformed into performance characteristics and resulted in economic parameters. Some bio-economic specifics of the respective resilience indicators are therefore also worth mentioning here.

#### 4.2.1. Milk Production

Regarding the performance parameters included in the current bio-economic evaluation of resilience indicators, milk production traits (yield, persistence, quality, SCS) were considered in as much detail as possible (mean and variability represented by standard deviation, as well as lactation order specifics shown in Table 2, Table 3, Table 4 and Table 5 and Figure 2 and Figure 3). The lower milk yield in more resilient cows found in our study has also been predicted in genetic terms, with the aim of guiding selection to improve resilience [30]. As milk is the core source of revenues for dairy farms [27], this explains its dominant position in terms of profit change found in our study (43% on average, ranging from 17% to 56% (Figure 4)). Similarly, its importance was shown in the higher profitability of less resilient cows (Q1) compared to those with higher resilience (Q3) across all indicators. Improved profitability probably resulted from higher milk yield and thus lower costs per kilogram produced (−6%, on average). This corresponds to the findings of the authors of [27], who observed improved profitability on high-producing farms due to lower unit costs.

Additionally, the dominant position of milk could be related to resilience indicator determination itself, as milk yield variability was taken as the basis. Nevertheless, some indicator specifics appeared when slightly lower (17% and 38%, respectively) influences on profit change were found for Q3 and Q1 cows in the r-auto indicator. These results supported the character of the r-auto indicator, which is known to be weakly associated with milk yield and to capture a specific aspect of resilience (length of recovery), whereas the associations of other indicators with milk yield are stronger, considering vulnerability to short-term perturbations [4,8,9].

#### 4.2.2. Longevity

Simultaneously, in the context of r-auto, the relative proportion of longevity’s influence on farm profit change was larger (38% to 44%) compared with other indicators (7% to 33%). The special position of longevity in r-auto can be seen in the context of its average value among quartiles. The longest lifetime was found for cows in the median (Q2) group in r-auto, whereas it was Q3 cows with the Var and LnVar indicators. In spite of the fact that better resilience is generally associated with greater longevity (Q3 in Var and LnVar in our study (see also [4,7])), this was true for the Q2 group when using the r-auto indicator. This suggests that cows with r-auto resilience values close to zero (Q2 group) are more favorable in terms of longevity and overall system profitability while maintaining milk yield at the average level. This finding corresponds to the hypothesis of Berghof et al. [2], who mentioned that more resilient animals are those with r-auto closer to zero. The breeding value definition (for which the r-auto resilience indicator is desirable) should be scrutinized.

Simulating cow longevity in the bio-economic model, the mutual proportions of reasons for cow loss (low milk yield, health, and reproduction problems) remained the same among the evaluated quartile datasets. This ratio can be modified in the input file (in the case of sufficient data) to take into account their specific participation in lifetime and associated revenues and costs. In terms of other performance and economic parameters, a nonlinear relationship between longevity and profitability, as found by Adamie et al. [28], could become more visible. In our study, cows with the shortest lifetimes (2.5 lactations) and highest milk yields (11,533 kg per 305 d period, both in Q1 LnVar) reached one of the highest profitability levels (3.3%) among the evaluated quartiles. The relative complexity of longevity and associated performance parameters was shown in our economic evaluation.

#### 4.2.3. SCS, Health, and AFC

For SCS and AFC, low proportional representation was found in the profit change in resilient indicators (means of 1% for both). In the case of SCS, we considered the indirect effect of udder health on milk price. Regarding the present payment system and SCS, approximately 98% and 99% of the milk produced by cows in groups Q2 and Q3, respectively, represent the first class and correspond to the economic changes found. In cases where the direct health parameters (e.g., mastitis, hoof health, and reproduction) of the studied resilience groups are known, their economic impacts can be considered and may become more visible in terms of health outcomes. Indeed, a favorable relationship between resilience and health traits has been reported [4,9]. Secondly, veterinary costs, which were held constant in the evaluated resilience groups, could be adjusted according to the relevant data. Special inputs for such assessment have already been included in the bio-economic model [11]. The economic consequences of health (disease incidence and veterinary costs) among the studied resilience indicators will probably be more intense than with SCS as a function of milk price. The same can be said for AFC when the heifer live weight and conception rate specifics among resilience groups are known (as mentioned above). AFC’s contribution to the farm profit change would probably be slightly greater, taking the change in costs and revenues of individual resilience groups into account more comprehensively.

#### 4.2.4. Feed Intake and Efficiency

Further potential resilience-related characteristics of production systems could be represented by feed intake [1] and quality/composition [4]. Lower feed consumption and animal production might be expected in less resilient animals. Feed intake (variability) and feeding duration are presumed resilience measures, mainly in meat production systems [2]; this is similar to the way milk yield variability may be applied to dairy cattle.

Nevertheless, feed intake and quality (nutritional value) were considered in our study, and related feed costs were calculated in the bio-economic model [11] to cover the nutrition (energy and protein) requirements for the maintenance and production (growth, pregnancy, milk yield and quality) of the given animal category and resilience groups. Based on our results (Table 2), cows that produced less milk with lower DMY deviations from the predicted lactation curve were identified as more resilient (group Q3). Lower milk production (−369 kg/305 days in milk) and thus lower feed costs (−EUR 31/cow in second parity) were calculated for these cows (Q3 vs. Q2 group, on average). Therefore, feed efficiency (expressed as kilograms of milk produced per kilogram of fresh feed consumed) varied among resilience quartiles across all indicators (e.g., 0.80 kg vs. 0.88 kg of milk in Q3 vs. Q1 cows in second lactation in the Var indicator). However, according to Bengtsson et al. [30], a different reallocation of resources should also be considered in this case. In more resilient cows (Q3 group), fewer milk yield resources can be assigned for reproduction (e.g., manifested in shorter insemination intervals in our dataset), body reserves (due to better body condition score, as found by Bengtsson et al. [30]), or to deal with disturbances (better resilience). Finally, as mentioned above, such energy allocation can support the longevity of more resilient cows (Var and LnVar in our study [7,30]). In our economic evaluation, due to the absence of detailed information, cows’ body condition score (represented by live weight) remained constant among the studied quartiles. Live weight is already taken as an input value (to calculate the associated requirements for heifer growth and cow maintenance), but could easily be altered according to resilience quartile when needed.

Bengtsson et al. [30] further suggested that feed efficiency should be evaluated over cows’ lifetimes, expecting higher lifetime lactation efficiency in animals with higher resiliency. When considering higher production longevity in our economic evaluation, feed efficiency (i.e., milk yield/fresh feed unit) was still lower (−7%) and total lifetime costs per unit of milk remained slightly higher (+6%) for more vs. less resilient cows. Nevertheless, this expectation might be met if the feed needed to maintain heavier cows in the Q3 group or the change in other cost items mentioned above (e.g., veterinary) in more resilient cows were taken into account. Another option is to directly alter the feed efficiency among resilience groups. The economic importance of cows’, heifers’, and fattened animals’ residual feed intake is already integrated into the bio-economic model [11]. Shifting the daily feed intake of cows by −0.05 kg of dry matter while maintaining the same production level would enhance the annual farm profit by EUR 84/cow (varying by ±EUR 1 among resilience indicator groups), representing potential savings due to improved feed efficiency.

#### 4.2.5. Further System Associations

Generally speaking, additional cost items (personnel and fixed costs, depreciation) could be adjusted among resilience indicator groups, provided that data are available. Likewise, various production system characteristics (e.g., conventional vs. organic, differences in milking technology, and farm size), which influence the values of inputs (feeds, medicaments, fixed costs, and depreciation) and outputs (milk, meat, and animal prices), and therefore farm profitability, could be considered in bio-economically evaluating animal resilience indicators. For example, some personnel time savings could be expected when animals are healthy and easy to manage [2] and organic farms could be less productive in terms of milk output when compared with conventional farms [28]. Furthermore, larger farms can achieve higher profitability [27,28], likely due to economies of scale. Nevertheless, they could have greater animal welfare challenges and require stronger animal resilience [2].

In the context of the production system, some specifics of more resilient animals should also be considered. Some of the dairy farm benefits found in our study, such as higher milk quality, udder health, herd turnover, and longevity, were similar to the better heifer fertility and calf survival reported for beef cattle [31]. Furthermore, the associations between indicators and production parameters (milk yield in our study and live weight in beef cattle [31]) were mostly unfavorable. When comparing performance and resilience, some specific aspects of resilience indicators (LnVar and Var vs. r-auto) appeared in both populations. Animals selected for higher milk yield (dairy) and live weight (beef) are less resilient to short-term perturbations but showed faster recovery [4,8,31]. Therefore, some compensation could be expected when selecting a combination of resilience indicators. Rodriguez et al. [31] further suggested that such an unfavorable association could generally be based on higher nutrition requirements for production and thus less resilience to environmental perturbations. Therefore, it could be suggested that averagely resilient animals maintain their performance and profitability, perhaps representing an optimal balance.

## 5. Conclusions

Our study combines cows’ relative breeding values for resilience indicators with their performance characteristics. The performance database represented a useful source of information, and the bio-economic model was confirmed as a suitable tool for the economical evaluation of indicator specifics. Animal resilience displayed the broad impact on herd structure, production, reproduction, costs, revenues, and overall farm economics. Prioritizing animals expressing better resilience would improve milk quality, udder health, herd turnover, and longevity along with lower milk yield offsetting the mentioned benefits. Milk yield, lactation persistence, longevity, and milk fat and protein content contributed most strongly to change in farm profit. Any use of a resilient indicator in animal selection should be attentive to the specifics of associated performance and economic changes. Animals with average resilience at least maintain their performance and associated profitability, and could represent an optimal balance. Similarly, selecting a combination of resilience indicators could provide partial compensation for the immediate change in performance by achieving faster recovery from environmental instabilities. A comprehensive evaluation of resilience and farm economics revealed further systemic associations that could facilitate an even deeper examination of resilience and detailed data for knowledge-based farm management and decision making.

## Figures and Tables

**Table 1:** Description of initial datasets ^1^ and resilience indicators ^2^ for overall data and quartiles.

**Initial Dataset**										
**Parameter**		**N**		**Mean**		**SD**		**Min/Max**		
DMY (kg per day)										
Conventional		278,862		38.11		9.34		2.00/95.40		
AMS		679,269		36.59		9.99		2.01/93.87		
Robotic parlor		202,088		39.00		7.70		2.00/78.88		
Total		1,160,219		37.34		9.52		2.00/95.40		
DIM (days)		1,160,219		148.61		87.39		0.00/350.00		
**Resilience Indicators**										
**Data Group**		**Var**			**LnVar**			**r-auto**		
		**N**	**Mean**	**SD**	**N**	**Mean**	**SD**	**N**	**Mean**	**SD**
Overall dataset		3655	97.68	17.34	3655	97.25	16.08	3655	99.38	12.92
Resilience indicator quartile	Q3	911	109.34	8.20	912	108.52	11.45	914	108.13	13.80
	Q2	1827	97.69	17.34	1824	98.19	16.08	1826	99.61	12.92
	Q1	917	86.43	9.20	919	86.58	14.10	915	91.03	15.94

^1^ DMY—daily milk yield (kg); AMS—automatic milking system; DIM—days in milk; N—number; mean, min, max—average, minimum, and maximum values; SD—standard deviation. ^2^ Quartiles: Q3—25% most resilient cows; Q2—median; and Q1—25% least resilient cows, according to their relative breeding values estimated for the following resilience indicators: Var—natural log-transformed variance of daily milk yield; LnVar and r-auto—natural log-transformed variance and lag-1 autocorrelation of daily deviations of observed milk yields from the predicted individual lactation curve, respectively. Based on the authors’ calculations from the database provided by CMBC, Inc. Prague, Czech Republic.

**Table 2:** Basic phenotypic parameters and their characteristics ^1^ for the overall dataset and for resilience indicator quartiles (Q) ^2^.

Data Group			Parameter (Unit) ^1^											
			MY (kg)	FY (kg)	PY (kg)	SCC (ths./mL)	PER (%)	LD (Days)	II (Days)	SP (Days)	CI (Days)	AFC (Days)	CUL (Days)	LONG (Lact.)
Overall dataset		mean	11,192	410	378	190	97	327	75	107	379	751	1816	2.81
		SD	1971	47.80	43.54	14.40	12.19	67.76	19.99	42.19	46.66	93.74	622.14	1.42
Var	Q3	mean	10,740	403	370	174	98	334	75	108	379	753	1860	2.91
		SD	2174	48.49	43.67	14.33	9.75	70.88	19.27	42.47	44.18	96.61	647.14	1.47
	Q2	mean	11,261	412	380	186	97	328	75	107	380	752	1775	2.72
		SD	2204	47.80	43.35	14.38	11.67	66.13	19.06	42.02	48.52	94.62	628.56	1.44
	Q1	mean	11,504	413	381	214	98	320	76	104	378	748	1763	2.66
		SD	2254	46.90	43.49	14.49	15.11	67.17	22.38	42.24	45.21	89.00	539.13	1.27
LnVar	Q3	mean	10,783	403	372	173	96	331	75	108	382	757	1910	3.02
		SD	2119	47.37	42.15	14.33	11.19	65.64	20.01	43.14	45.72	97.92	623.36	1.49
	Q2	mean	11,225	411	379	184	98	328	75	106	379	752	1844	2.88
		SD	2221	48.25	44.05	14.38	12.06	67.53	19.06	41.51	47.68	95.40	631.57	1.44
	Q1	mean	11,533	415	381	219	97	324	75	106	377	745	1689	2.50
		SD	2278	47.13	43.74	14.51	13.33	70.13	21.72	42.59	45.68	85.50	583.32	1.30
r-auto	Q3	mean	11,048	410	377	165	98	327	75	105	375	755	1739	2.63
		SD	2303	51.01	45.40	14.30	11.54	65.92	19.04	42.01	42.43	93.90	562.37	1.29
	Q2	mean	11,191	410	378	199	97	329	76	108	382	752	1868	2.92
		SD	2187	46.14	42.88	14.43	12.27	69.46	20.92	42.55	47.60	95.33	651.31	1.50
	Q1	mean	11,337	411	378	196	97	325	75	105	378	746	1776	2.72
		SD	2219	47.80	42.99	14.42	12.65	66.08	19.01	41.65	48.30	90.12	603.65	1.37

^1^ Mean and standard deviation (SD) for phenotypic parameters: MY, FY, and PY—milk, fat, and protein yield per 305 days of milking; SCC—somatic cell count; PER—lactation persistence; LD—number of days in lactation; II—insemination interval; SP—service period; CI—calving interval; AFC—age at first calving; CUL—age at culling; and LONG—number of lactations ((CUL − AFC)/CI). ^2^ Quartiles: Q3—25% most resilient cows; Q2—median; and Q1—25% least resilient cows, according to their relative breeding values estimated for the following resilience indicators: Var—natural log-transformed variance of daily milk yield; LnVar and r-auto—natural log-transformed variance and lag-1 autocorrelation of daily deviations of observed milk yields from the predicted individual lactation curve, respectively. Based on the authors’ calculations from the database provided by CMBC, Inc.

**Table 3:** Lactation curve ^1^ parameters in respective lactations for the overall dataset and resilience indicator quartiles ^2^.

Data Group		Parameter of Lactation Curve/Lactation							
		a		b			c		
		1	2+	1	2	3+	1	2	3+
Overall dataset		30.108	35.323	0.01225	0.07340	0.08610	0.000210	−0.001631	−0.002025
Var	Q3	28.858	34.073	0.01200	0.06782	0.07320	0.000072	−0.001389	−0.001650
	Q2	30.142	35.357	0.01230	0.08080	0.08910	0.000183	−0.001771	−0.002081
	Q1	30.378	35.593	0.02110	0.07725	0.10149	0.000265	−0.001806	−0.002401
LnVar	Q3	28.858	34.073	0.01059	0.07150	0.09132	0.000160	−0.001688	−0.002140
	Q2	30.108	35.323	0.00815	0.07780	0.08490	0.000312	−0.001678	−0.001955
	Q1	30.885	36.100	0.01900	0.07070	0.09118	0.000092	−0.001541	−0.002160
r-auto	Q3	29.466	34.681	0.00480	0.08040	0.08715	0.000402	−0.001705	−0.001944
	Q2	30.074	35.289	0.00950	0.07552	0.08732	0.000278	−0.001688	−0.002085
	Q1	30.176	35.391	0.03290	0.06969	0.08950	−0.000219	−0.001553	−0.002065

^1^ Lactation curve based on Wood function [17] as modified by [18] for the first (1), second (2), second and higher (2+), and third and higher (3+) lactations. The parameter *d* was set to −0.0000001 for all dataset groups and lactations. ^2^ Quartiles: Q3—25% most resilient cows; Q2—median; and Q1—25% least resilient cows, according to their relative breeding values estimated for the following resilience indicators: Var—natural log-transformed variance of daily milk yield; LnVar and r-auto—natural log-transformed variance and lag-1 autocorrelation of daily deviations of observed milk yields from the predicted individual lactation curve, respectively. Based on the authors’ calculations from the database provided by CMBC, Inc.

**Table 4:** Additional phenotypic parameters and their characteristics ^1^ for the overall dataset and resilience indicator quartiles (Q) ^2^.

Data Group			Parameter (Unit) ^1^				
			Fat Content (FC; %)	Protein Content (PC; %)	Somatic Cell Score (SCS; Score)	Days in Dry (Day)	Pregnancy Length (Day)
Overall dataset		mean	3.69	3.39	3.92	52	273
		SD	0.224	0.090	0.204	10.76	33.55
Var	Q3	mean	3.78	3.45	3.80	45	272
		SD	0.229	0.092	0.197	9.66	31.65
	Q2	mean	3.69	3.38	3.89	52	273
		SD	0.223	0.090	0.202	10.58	34.85
	Q1	mean	3.62	3.32	4.09	58	273
		SD	0.219	0.089	0.213	12.10	32.72
LnVar	Q3	mean	3.76	3.46	3.79	51	274
		SD	0.228	0.092	0.197	10.11	32.83
	Q2	mean	3.69	3.38	3.88	52	273
		SD	0.224	0.090	0.202	10.67	34.32
	Q1	mean	3.62	3.31	4.13	54	271
		SD	0.219	0.0885	0.215	11.63	32.83
r-auto	Q3	mean	3.73	3.42	3.72	48	270
		SD	0.226	0.091	0.194	9.73	30.52
	Q2	mean	3.69	3.39	3.99	53	274
		SD	0.224	0.090	0.207	11.22	34.16
	Q1	mean	3.65	3.35	3.97	54	273
		SD	0.221	0.089	0.206	10.89	34.85

^1^ SD—standard deviation. ^2^ Quartiles: Q3—25% most resilient cows; Q2—median; and Q1—25% least resilient cows, according to their relative breeding values estimated for the following resilience indicators: Var—natural log-transformed variance of daily milk yield; LnVar and r-auto—natural log-transformed variance and lag-1 autocorrelation of daily deviations of observed milk yields from the predicted individual lactation curve, respectively. Based on the authors’ calculations from the database provided by CMBC, Inc.

**Table 5:** Statistical differences ^1^ between phenotypic parameters ^2^ in the studied resilience indicator quartiles ^3^.

Data Group		MY (kg)	FY (kg)	FC (%)	PY (kg)	PC (%)	SCS (Score)	PER (%)	LD (Days)	II (Days)	SP (Days)	CI (Days)	AFC (Days)	CUL (Days)	LONG (Lact.)
Var	Q1:Q2	**0.001**	0.992	**0.001**	0.872	**0.001**	**0.001**	0.950	**0.009**	**0.035**	0.495	0.512	0.890	0.313	0.281
	Q2:Q3	**0.001**	**0.044**	**0.001**	**0.001**	**0.001**	**0.001**	0.134	**0.025**	0.505	0.447	0.994	0.899	**0.014**	**0.002**
	Q1:Q3	**0.001**	0.094	**0.001**	**0.001**	**0.001**	**0.001**	0.307	**0.001**	**0.020**	0.851	0.571	0.818	0.170	0.060
LnVar	Q1:Q2	**0.001**	0.624	**0.001**	0.790	**0.001**	**0.001**	0.230	0.223	**0.045**	0.459	0.968	0.317	**0.038**	**0.011**
	Q2:Q3	**0.001**	**0.010**	**0.001**	**0.002**	**0.001**	**0.001**	**0.022**	**0.022**	0.292	0.720	0.787	0.396	0.180	0.481
	Q1:Q3	**0.001**	**0.010**	**0.001**	**0.005**	**0.001**	**0.001**	0.501	0.501	**0.010**	0.729	0.836	0.921	**0.004**	**0.007**
r-auto	Q1:Q2	0.203	0.702	**0.002**	0.503	**0.001**	**0.008**	**0.023**	**0.009**	**0.035**	0.495	0.512	0.553	0.313	0.281
	Q2:Q3	**0.047**	0.642	**0.039**	0.887	**0.001**	**0.010**	0.584	**0.025**	0.505	0.851	0.994	**0.030**	**0.014**	**0.002**
	Q1:Q3	**0.005**	0.355	**0.001**	0.408	**0.001**	**0.001**	**0.014**	**0.001**	**0.020**	0.447	0.571	0.164	0.170	0.060

^1^ Significant *p*-values (*p* < 0.05) are in bold font. Statistical differences calculated by ANOVA for unbalanced data using the GLM procedure of SAS 9.4 (SAS Institute, Cary, NC, USA). ^2^ Phenotypic parameters: MY, FY, and PY—milk, fat, and protein yield per 305 days in milk; SCC—somatic cell count; PER—lactation persistence; LD—number of days in lactation; II—insemination interval; SP—service period; CI—calving interval; AFC—age at first calving; CUL—age at culling; and LONG—number of lactations ((CUL − AFC)/CI). ^3^ Quartiles: Q3—25% most resilient cows; Q2—median; and Q1—25% least resilient cows according to their relative breeding values estimated for the following resilience indicators: Var—natural log-transformed variance of daily milk yield; LnVar and r-auto—natural log-transformed variance and lag-1 autocorrelation of daily deviations of observed milk yields from the predicted individual lactation curve, respectively. Based on the authors’ calculations from the database provided by CMBC, Inc.

**Table 6:** Economic parameters of the studied resilience groups ^1^ and their differences ^2^ from the respective Q2 group median and overall dataset mean (in brackets).

Data Group ^1^		Economic Parameters				Difference ^2^ from the Respective Resilience Q2 Group (Overall Dataset)						
		Revenues	Costs	Profit	Profitability	Revenues		Costs		Profit		Profitability
		EUR per Cow and per Year			%	EUR	(%)	EUR	(%)	EUR	(%)	p.p.
Overall dataset		4643	4507	136	3.0	–	–	–	–	–	–	–
Var	Q3	4641	4526	115	2.5	−28 (−2)	−1 (0)	10 (19)	0 (0)	−38 (−21)	−25 (−15)	−0.9 (−0.5)
	Q2	4669	4516	153	3.4	(26)	(1)	(8)	(0)	(18)	(13)	(0.4)
	Q1	4626	4498	128	2.9	−43 (−17)	−1 (0)	−18 (−10)	0 (0)	−25 (−7)	−16 (−5)	−0.5 (−0.2)
LnVar	Q3	4673	4546	128	2.8	40 (30)	1 (1)	44 (38)	1 (1)	−4 (−8)	−3 (−6)	−0.1 (−0.2)
	Q2	4633	4502	132	2.9	(−10)	(0)	(−6)	(0)	(−4)	(−3)	(−0.1)
	Q1	4649	4502	146	3.3	15 (6)	0 (0)	1 (−5)	0 (0)	15 (11)	11 (8)	0.3 (0.2)
r-auto	Q3	4623	4508	115	2.6	−54 (−20)	−1 (0)	−16 (1)	0 (0)	−37 (−20)	−25 (−15)	−0.8 (−0.5)
	Q2	4677	4524	153	3.4	(34)	(1)	(17)	(0)	(17)	(13)	(0.4)
	Q1	4612	4490	122	2.7	−64 (−30)	−1 (−1)	−34 (−17)	−1 (0)	−30 (−13)	−20 (−10)	−0.6 (−0.3)

^1^ Overall dataset parameters and quartiles: Q3—25% most resilient cows; Q2—median; and Q1—25% least resilient cows, according to their relative breeding values estimated for the following resilience indicators: Var—natural log-transformed variance of daily milk yield; LnVar and r-auto—natural log-transformed variance and lag-1 autocorrelation of daily deviations of observed milk yields from the predicted individual lactation curve, respectively. ^2^ Difference expressed in absolute value (EUR per cow and per year; p.p.—percentage points) and in relative terms (%). Based on the authors’ calculations using the EWDC program [11] and the data provided by CMBC, Inc. An exchange rate of 25.119 CZK/EUR was applied (https://www.kurzy.cz/kurzy-men/historie/EUR-euro/2024/; accessed on 24 June 2025).

## Data Availability

The initial data used in this study are the property of the farmers and Czech and Moravian Breeders’ Corporation, Inc. and therefore cannot be publicly shared.

## Supplementary Materials

The following supporting information can be downloaded at https://www.mdpi.com/article/10.3390/ani15243593/s1. Table S1: Performance parameters relevant for the overall dataset set up in the bio-economic model (input–output mapping).
