# Supplementary material for: A Bio-Economic Evaluation of Var, LnVar, and r-Auto Resilience Indicators in Czech Holstein Cattle
Source: Animals (Basel). 2025 Dec 14;15(24):3593. doi: 10.3390/ani15243593 (PMC12729534; doi:10.3390/ani15243593)
Supplement: Supplementary file 1 [file animals-15-03593-s001.zip › animals-4008017-supplementary.pdf]

animals

MDPI

Supplement

A Bio-Economic Evaluation of Var, LnVar, and r-Auto Resilience Indicators in Czech Holstein Cattle

Zuzana Krupová *, Eva Kašná, Ludmila Zavadilová and Emil Krupa

Table S1. Performance parameters relevant for the overall dataset set up in the bio-economic model (input–output mapping)[1].

**Blocks of performance parameters**
**1. herd turnover**
INPUT11
Gestation length (days): 272.8000
Average interval between calving and first insemination (days): 75.2000
Number of days dry (days): 52.0000
RESULT FILE
Average calving interval (days): 379.43
CHECKD
Length of the whole lactation in reproductive cycle i + 1 (i = 0, ...,LL − 1)
(difference between the length of the calving interval and days dry)
dayslac[0] = 327.435364
dayslac[1] = 327.417725
dayslac[2] = 327.417419
dayslac[3] = 327.418762
dayslac[4] = 327.418060
dayslac[5] = 327.418213
dayslac[6] = 327.416901
dayslac[7] = 327.416901
Average cow service period in days: avsp = 106.625656
**2. age of heifers entering herd (age at first calving)**
INPUT15, INPUT07, INPUT11
Daily gain of female calves from birth till the end of the rearing period (kg/day): 0.7990
Daily gain of breeding heifers from the end of the rearing period to 1st mating (kg/day): 0.7790
Weight of heifers at 1st mating (kg): 400.0000
Vector of conception rate after ith insemination for heifers (i=1 to inmaxh where inmaxh is the maximal number of inseminations for heifers) ( ): 0.5930 0.5760 0.5500
Maximal number of inseminations per heifer ( ): 3
RESULT FILE
Average age at 1st calving (days): 751.4
**3. lifetime - cow herd structure**
INPUT07

 https://doi.org/10.3390/10.3390/ani15243593

Vector of cow losses within reproductive cycles 1 to LL as proportion of cows entered the reproduction cycle

( ): 0.0613 0.0330 0.0990 0.0830 0.1380 0.0860 0.1120 0.0800

Vector of cows culled within reproductive cycles 1 to LL for health problems other than dystocia as proportion of cows entered the reproduction cycle. Cows culled for failure to conceive must not be included.

( ): 0.0683 0.1680 0.1490 0.1230 0.1780 0.1560 0.1620 0.1200

Vector of cows culled within reproductive cycles 1 to LL for low milk production as proportion of cows entered the reproduction cycle

( ): 0.0433 0.0330 0.0990 0.1030 0.1580 0.1460 0.1320 0.0400

RESULT FILE

Probabilities that a cow is in the given reproductive cycle

Reproductive cycle 1: 0.3948

Reproductive cycle 2: 0.2695

Reproductive cycle 3: 0.1690

Reproductive cycle 4: 0.0867

Reproductive cycle 5: 0.0482

Reproductive cycle 6: 0.0188

Reproductive cycle 7: 0.0090

Reproductive cycle 8: 0.0041

Sum: 1.0000

Average lifetime of cows in number of calvings: 2.53

4. udder health – SCS

INPUT28

Mean of somatic cell score in the dairy cow population ( ): 3.9200

Phenotypic standard deviation of bulk tank somatic cell score in the dairy cow population ( ): 0.2040

5. milk quality - fat and protein content

INPUT11, INPUT28

Fat content in milk (%): 3.6900

Protein content in milk (%): 3.3900

Standard deviation for milk fat content (%): 0.2240

Standard deviation for milk protein content (%): 0.0904

6. milk production and persistence

INPUT22

Average milk yield per cow and year (kg): 9820.0000

Parameter b for the first lactation ( ): 0.0122

Parameter b for the second lactation ( ): 0.0734

Parameter b for third and higher lactations ( ): 0.0861

Parameter c for the first lactation ( ): 0.0002

Parameter c for the second lactation ( ): -0.0016

Parameter c for the third and higher lactations ( ): -0.0020

Parameter d for the first lactation ( ): -0.0000

Parameter d for the second lactation ( ): -0.0000

Parameter d for the third and higher lactations ( ): -0.0000

CHECKD

305-d milk yield averaged over lactations milk305ave = 11233.84

305d fat yield (kg)	fat305ave =	414.53
305d protein yield (kg)	prot305ave =	380.83

[1] Performance parameters were set up in the input (INPUTxx) and output files (result file, CHECKD) of the bio-economic model of the program EWDC (Wolf et al. 2023) in the following blocks: 1) herd turnover, 2) age of heifers entering herds, 3) lifetime, 4) udder health, 5) milk quality, and 6) milk production and persistence. Values represent the overall dataset performance (see Tables 2 to 4 and Figures 1 and 2). Based on authors' calculations from the database provided by CMBC, Inc.
